# Gene expression knowledge graph for patient representation and diabetes prediction

**DOI:** 10.1186/s13326-025-00325-6

**Published:** 2025-03-08

**Authors:** Rita T. Sousa, Heiko Paulheim

**Affiliations:** Data and Web Science Group, University of Mannheim, 68159 Mannheim, Germany

**Keywords:** Diabetes prediction, Expression data, Knowledge graph, Ontology, Knowledge graph embedding, Representation learning

## Abstract

Diabetes is a worldwide health issue affecting millions of people. Machine learning methods have shown promising results in improving diabetes prediction, particularly through the analysis of gene expression data. While gene expression data can provide valuable insights, challenges arise from the fact that the number of patients in expression datasets is usually limited, and the data from different datasets with different gene expressions cannot be easily combined. This work proposes a novel approach to address these challenges by integrating multiple gene expression datasets and domain-specific knowledge using knowledge graphs, a unique tool for biomedical data integration, and to learn uniform patient representations for subjects contained in different incompatible datasets. Different strategies and KG embedding methods are explored to generate vector representations, serving as inputs for a classifier. Extensive experiments demonstrate the efficacy of our approach, revealing weighted F1-score improvements in diabetes prediction up to 13% when integrating multiple gene expression datasets and domain-specific knowledge about protein functions and interactions.

## Introduction

Diabetes is a chronic health condition resulting from insufficient insulin production by the pancreas or the body’s inability to utilize the insulin it generates effectively [1]. This disease has emerged as a worldwide health issue, impacting millions of people globally. According to the World Health Organization, in 2019, diabetes directly contributed to 1.5 million deaths, with 48% occurring before the age of 70. Besides that, this chronic disease is associated with the development of several comorbidities, such as blindness, kidney failure, heart attacks, strokes, and lower limb amputation.

Due to the multidisciplinary nature of diabetes, predicting and detecting this complex disease continues to pose a significant challenge. In the last decades, some approaches have demonstrated encouraging outcomes using machine learning methods to identify patterns and potential risk factors linked to diabetes, allowing not only the early detection of diabetes but also enabling tailored interventions [2–5]. These machine learning approaches encompass several types of data, including electronic health records [6], imaging data [7], and demographic data [8]. Omics data, namely gene expression datasets, have also received attention since genomics, epigenomics, and transcriptomics can help understand the critical pathways and regulatory mechanisms in diabetes [9].

While gene expression datasets are readily accessible in public databases, and gene expression analysis is a powerful tool for pinpointing genes associated with diseases, particularly in the context of diabetes prediction, a significant issue arises in handling this type of data. On the one hand, gene expression datasets often exhibit a limitation in the number of patients, with a relatively small number of included patients. Conversely, supervised machine learning methods are data-driven, relying on a large number of labeled data for effective training and performance. One alternative involves combining multiple expression datasets to increase the patient pool for training machine learning models. However, this brings us to the challenge of how to integrate the information about multiple expression datasets, as each dataset may measure gene expression across distinct genes. Additionally, variations in experimental platforms and designs across different studies further complicate integration efforts. These challenges highlight the limitations of current approaches based on gene expression data. Those approaches either focus on a single dataset, limiting the scope of their analysis, or attempt to integrate data from multiple datasets, but this integration is often constrained to using the expression values for a set of common genes. The latter approaches reduce the comprehensiveness of the analysis and may overlook valuable information from genes present in one dataset but absent in others. Furthermore, they fail to adequately consider the complex relationships and interactions that exist between genes, which are critical for understanding the underlying biological processes. Consequently, there is a need for a solution that addresses the limitations of single dataset analysis, accommodate the diversity across different datasets, and incorporate gene interactions into the overall framework.

Knowledge graphs (KGs) present a unique and promising solution. KGs can represent knowledge about concepts and relationships in a fully machine-readable format [10]. Moreover, several biomedical ontologies are publicly available to enrich KGs [11], enabling the representation of domain-specific knowledge. In fact, over the past few years, biomedical ontologies and KGs have emerged as a tool for biomedical data integration and have been adopted in many machine learning applications, with KG embedding approaches [12] becoming increasingly popular [13]. KG embedding approaches transform entities and relationships in a KG into a lower-dimensional vector space while attempting to preserve the graph structure and, in some cases, semantic information. An alternative solution that has gained significant attention in recent years involves the use of graph neural network (GNN) architectures explicitly designed for graph structures. However, these architectures are not well-suited for the inherently heterogeneous nature of KGs [10], particularly those enriched with ontological information. Additionally, GNNs typically require the presence of node features, which limits their applicability in biomedical scenarios where such features may not be available [14].

This work tackles the challenge of integrating heterogeneous gene expression datasets in biomedical applications, focusing on diabetes prediction. We propose a novel approach that generates a KG to incorporate both gene expression data and domain-specific knowledge and then employs KG embedding methods to generate vector representations of patients using different strategies. These patient representations serve as the input for a clustering method and a classifier to predict the likelihood of a patient having diabetes. We conduct an extensive evaluation of the impact of integrating multiple gene expression datasets comparing different strategies and KG embedding methods. The results show that incorporating other expression datasets and domain-specific knowledge improves diabetes prediction, emphasizing the efficacy of our approach.

## Related work

### Diabetes prediction using gene expression data

Several works have been using gene expression data to predict diabetes, employing diverse methodologies and datasets. In Li et al. [15], a support vector machine classifier is used for the diagnosis of diabetes. While multiple datasets were extracted from the Gene Expression Omnibus database, the machine learning model was trained on only one dataset, with three additional datasets used for validation. Feature selection involved the identification of ten common genes across all datasets. Mansoori et al. [16] and Kazerouni et al. [17] focus on long non-coding RNAs potentially associated with diabetes type 2. Both studies incorporated data collected from 100 diabetic and 100 non-diabetic subjects to train the classifiers. Mansoori et al. [16] employed logistic regression, whereas Kazerouni et al. [17] compare four classifiers (*K*-nearest neighbor, support vector machine, logistic regression, and artificial neural networks) to predict diabetes type 2 using the expression values for specific long non-coding RNAs as input. Both studies suggest that increasing the dataset with a larger number of patients would likely improve the performance of the classifiers. Furthermore, some other approaches explore expression data for diabetes prediction without employing machine learning methods [9, 18, 19].

### Integration approaches of omics data

With the growing collection of diverse molecular compartments, such as gene expression, DNA methylation status, and protein abundance, the volume of omics data has increased significantly, providing a unique opportunity to uncover biological mechanisms and pathways across diverse cell types. However, integrating omics data is challenging due to the varying dimensions across different data types (e.g., genes, proteins, metabolites), as well as differences in experimental conditions and sample types [20]. Therefore, several approaches have been proposed to facilitate the integration of multi-omics data. A possible solution to the integration problem is to map cells into a co-embedded space or non-linear manifold, allowing for the identification of shared features across cells within the omics space.

MultiMAP [21] is an approach for dimensionality reduction and integration that creates a non-linear manifold that represents different high-dimensional datasets. It normalizes distances within each dataset and between datasets based on specific neighborhood parameters. These distances are used to build a neighborhood graph on the manifold. Finally, MultiMAP projects both the manifold and the data into a shared low-dimensional embedding space by minimizing the cross-entropy between the graph in the manifold and the graph in the embedding space. GLUE (graph-linked unified embedding) [22] is a framework that uses a graph variational autoencoder to explicitly model regulatory interactions across omics layers, effectively bridging the gap between them. COBOLT [23] proposes a multimodal variational autoencoder based on a hierarchical Bayesian generative model to enable the joint analysis of cells across diverse omics datasets. StaBMap [24] is a mosaic data integration technique that constructs a topology based on shared features and subsequently maps cells to supervised or unsupervised reference coordinates by following the shortest paths within the topology. SIMBA (single-cell embedding along with features) [25] is a graph method that begins by representing different types of entities, such as cells and genes, into a single graph. For instance, if a gene is expressed in a particular cell, an edge is created between the gene and the cell, with the edge weight reflecting the gene’s expression level. Once the input graph is built, SIMBA utilizes a multi-relation graph embedding approach coupled with a Softmax-based transformation to project the nodes into a common low-dimensional space.

While these approaches have been proposed for integrating various types of omics data, some of them can also be applied to combining multiple gene expression datasets. However, our approach distinguishes by incorporating domain-specific knowledge, which allows capturing the relationships between genes both within individual datasets and across datasets.

### Knowledge graph embeddings

In the biomedical domain, the exploration of KGs has become increasingly prominent, with KG embedding methods emerging as particularly promising for capturing KG-based information [26]. These methods map entities and relationships in a KG into a lower-dimensional vector space while preserving graph structure and, in some cases, semantic information. Various types of KG embedding methods have been proposed to date.

Translational models, exemplified by TransE [27] and TransR [28] explore distance-based scoring functions. The basic idea of the translational distance models is that each fact represents the distance between the two entities, usually after a translation carried out by the relations. TransE is the most representative translational distance model, but several extensions have been introduced to address TransE limitations, namely TransR, which introduces a projection matrix for each relation.

On the other hand, semantic matching approaches, such as distMult [29], HolE [30], and ComplEx [31], use similarity-based scoring functions to capture the latent semantics of entities and relations in their vector space representations. DistMult takes the inherent structure of relations into account by employing tensor factorization. HolE combines the simplicity of DistMult with the power of RESCAL [32]. ComplEx extends DistMult by introducing complex embeddings to handle a large variety of binary relations.

Walk-based methods, such as RDF2vec [33], employ random walks to generate entity sequences as input to a neural language model that learns latent entity representations. Different walk-based approaches differ in their strategies for random walks and consideration of edge direction and type. In the context of biomedical KGs, characterized by rich hierarchical relations, walk-based approaches emerge as particularly well-suited, considering that these hierarchical relations can be more easily captured in walks.

## Methodology

Gene expression datasets typically only have few instances, and different datasets record different gene expressions. Thus, when training prediction models, one can either (1) use only one dataset, thereby having only little training data, or (2) try to combine multiple datasets. In the latter case, those are typically “incompatible” in the sense that they have different feature sets, i.e., a naive combination would lead to a larger dataset with lots of NULL values.

**Fig. 1:**
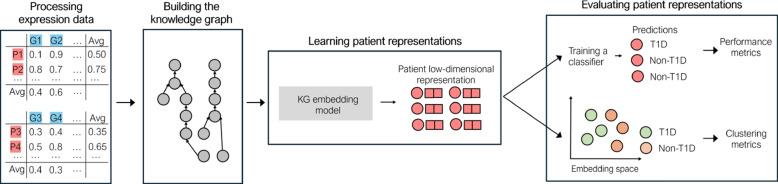
Overview of the proposed methodology with the main steps: processing gene expression data, building the KG, learning patient representations, evaluating patient representations

To overcome these challenges, we propose a methodology to integrate multiple expression datasets into a biomedical KG and then use it for diabetes prediction. Figure 1 shows an overview of this methodology. The first step corresponds to processing gene expression data. Then, the KG that integrates not only expression data from different datasets but also domain knowledge on protein function and protein interactions is built. The third step consists of generating a vector representation for each patient described in the biomedical KG. The last step involves evaluating the patient representations through diabetes prediction and distribution/ clustering of patient representations. The source code for our methodology is available on GitHub (https://github.com/ritatsousa/expressionKG).

### Expression data processing

Several studies have recently explored gene expression for diabetic and non-diabetic individuals, and the findings from these studies can be accessed in publicly available databases. The Gene Expression Omnibus (GEO) [34] is a public database maintained by the National Center for Biotechnology Information that archives high-throughput gene expression and other genomics datasets. Each GEO dataset represents a curated collection of biologically comparable GEO samples whose measurements are assumed to be calculated equivalently. The file associated with each dataset contains the raw gene expression data generated by microarrays. The data is structured in a tabular format, with each row corresponding to a unique patient, columns representing different gene fragments, and the cells containing specific expression values of those gene fragments for each respective patient.

Given the complexity of gene expression datasets, the pre-processing step is crucial. First, each probe of the microarray, identified by an identifier, contains a gene fragment for which the expression level is being determined. Each gene fragment is accompanied by an annotation detailing its biological context, indicating its association with a known gene. However, it is worth noting that not all gene fragments have such associations. Since our methodology relies on linking gene expression data with domain-specific knowledge describing gene functions, fragments without an associated gene are filtered out.

Another challenge involves the normalization of the expression values, as it helps to adjust the values within a specific range and improve comparability. In our work, we explore three alternatives for normalization: no normalization, gene normalization, and patient normalization. The first option, no normalization, leaves the data in its raw form. The second alternative, normalization of the values for each gene, adjusts the expression values across patients for each gene separately. The third alternative, normalization of the values for each patient, ensures that the gene expression values for each individual patient are scaled consistently. In both the gene and patient normalization methods, we apply a min-max scaling process. This involves subtracting the minimum value from each data point and dividing the result by the range (the difference between the maximum and minimum values). This transformation scales all values between 0 and 1, where the minimum value becomes 0, the maximum becomes 1, and all other values are proportionally adjusted within this range.

### Knowledge graph building

**Fig. 2:**
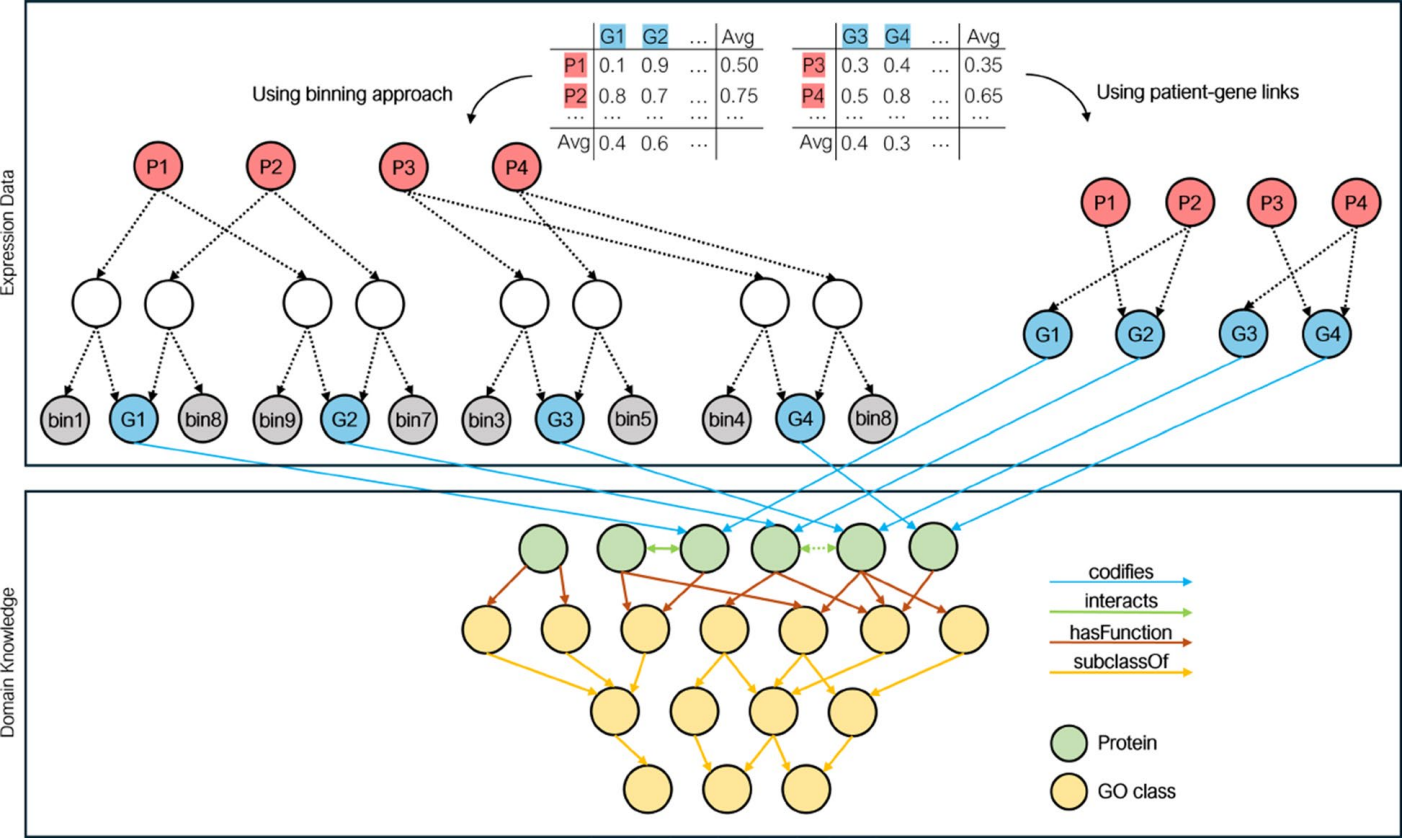
Schematic representation of how expression data and domain-specific knowledge are combined within the KG

The KG is built by integrating two types of data sources: expression data and domain-specific knowledge. Figure 2 illustrates the integration of the two sources into a KG. Since our approach relies on KG graph embeddings for generating patient representations and most embedding approaches are not able to handle numeric literals [35], we adopt two different strategies to include the expression data in the KG.

The first strategy involves representing patient gene expression values in a KG using a *binning approach*. Following the technique proposed in [35], we create bins from the set of expression values for each gene within a given dataset. The percentage of unique values defines the number of bins. For this strategy, we employed three options: using the non-normalized values, the gene-normalized values, or the patient-normalized values, depending on which normalization approach is applied to the expression data beforehand. This allows for flexibility in how the data is scaled before binning. To implement the binning strategy, a blank node is generated to represent the expression value attributed to a specific gene for a given patient. This establishes an association wherein a patient is connected to a blank node, which, in turn, is linked to a bin representing the expression value and the corresponding gene. Consider a simplified example using RDF where *_:x* denotes a blank node:

*(patientID, rdf:type, :Patient)*

*(:geneID, rdf:type, :Gene)*

*(:patientID, :hasExpression, _:x)*

*(_:x, :isExpressionOfGene, :geneID)*

*(_:x :hasValue :binID)*

The second strategy employs a *patient-gene links approach* based on expression values. A link between a patient and a gene is created when the patient’s expression value for that gene is higher than the calculated average expression value. For this strategy, two comparison options are available. The first is to compare the expression value of a particular gene in a patient with the patient’s average gene expression across all genes. The second option is to compare the expression value of that gene in the patient with the average expression of the same gene across all patients.

**Fig. 3:**
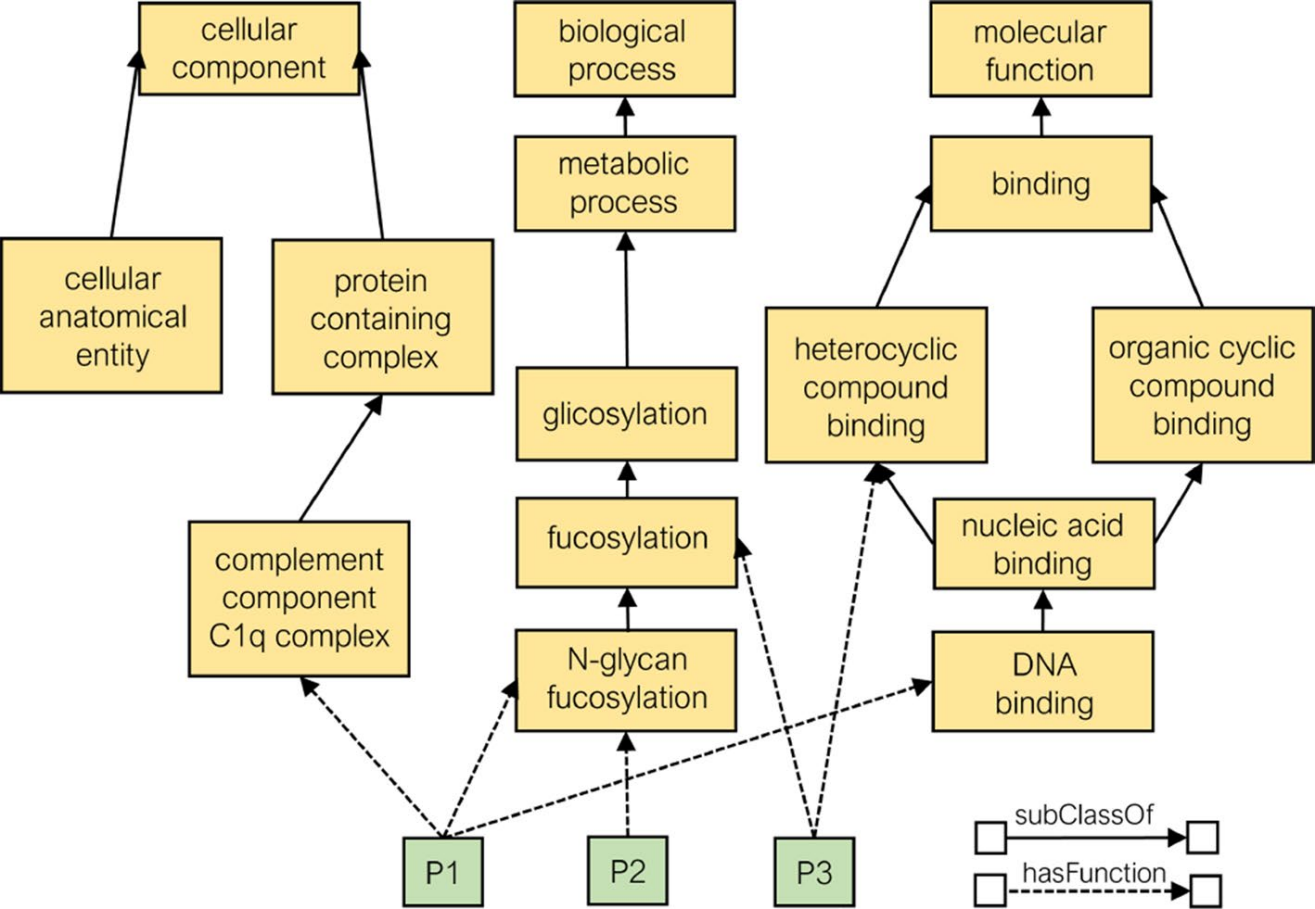
Subgraph of domain-specific knowledge

The domain-specific knowledge includes the Gene Ontology (GO) [36], GO annotation data [37], and protein-protein interaction (PPI) data [38]. The GO defines a hierarchy of classes that describe protein functions that can be represented as a graph where nodes are GO classes and edges define relationships between them. The GO encompasses three distinct domains for characterizing functions: the biological processes a protein is involved in, the molecular functions a protein performs, and the cellular components where a protein is located. These three domains of GO are represented as separate root ontology classes since they do not share any common ancestor. The GO annotation data refers to assigning functions represented as GO classes to proteins represented as links in the graph (see Fig. 3). Finally, the PPI data is extracted from STRING [38], one of the largest available PPI databases that integrates physical interactions and functional associations between proteins collected from several sources. Each interaction is scored based on its origin, with scores combined into a final value scaled between 0 and 1, reflecting STRING’s confidence in the biological relevance of the association. To ensure high-confidence interactions, only those with a combined score above 0.7 are included.

To bridge the gap between the two types of data sources, the expression data and the domain-specific knowledge, a gene in the expression data graph is mapped to a protein in the domain-specific KG. Online ID mapping tools, namely UniProt ID Mapping tool^1^, are used to convert identifiers between genes and proteins.

### Generating patient representations

We propose to generate patient representations by leveraging the information of multiple gene expression datasets and domain knowledge. As a preliminary step, the KG is converted into a directed and labeled RDF graph, following the W3C’s OWL to RDF Graph Mapping guidelines^2^. Next, our methodology employs six graph embedding approaches, namely RDF2vec, TransE, TransR, distMult, HolE, and ComplEx. These approaches were selected because they are representative of the main types of graph embedding techniques.

Two distinct approaches are employed to represent patients:

- The first approach involves generating KG embeddings directly for the patients using the KG. This approach was implemented across the multiple strategies for constructing the KG. These strategies include: (1) the binning approach without any normalization, (2) the binning approach with patient-level normalization, and (3) the binning approach with gene-level normalization. Additionally, two linking strategies were applied to establish links between patients and genes: one based on the average gene expression value for each patient, and the other using the average expression value of the gene across all patients.
- The second generates KG embeddings for the genes present in gene expression datasets and represents patients as the weighted average of gene embeddings, determined by the respective gene expression values. This approach can be applied independently of the specific strategy used to construct the KG. However, the weighted average used to represent patients can be calculated using either non-normalized gene expression values, patient-level normalized values, or gene-level normalized values.

In total, for each embedding method, 8 distinct representations of patients are employed. These representations result from the combination of several normalization strategies (non-normalized, patient-level normalization, and gene-level normalization), different strategies to build the KG (blank nodes and binning approach and inking approach between patients and genes based on expression values) and two approaches for representing patients with embeddings (direct patient embeddings and weighted average of gene embeddings). This diverse set of representations enables a comprehensive exploration of how various data preprocessing techniques, KG construction strategies, and embedding approaches affect the utilization of patient representations within the KG embedding space.

### Evaluating patient representations

We evaluate the different patient representations on two dimensions, diabetes prediction performance and distribution and clustering of patient representations, as shown in Fig. 1.

## Results and discussion

### Experimental setup

**Fig. 4:**
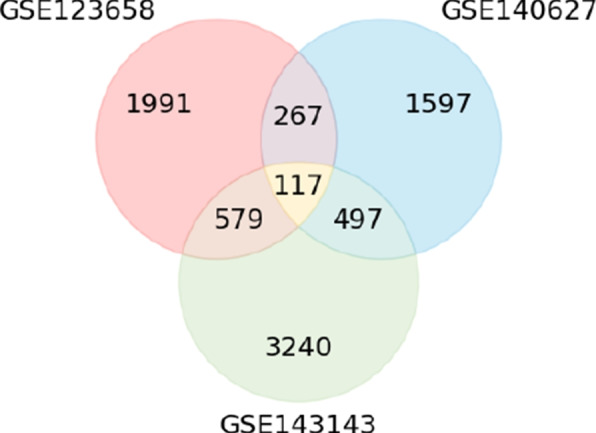
Venn diagram showing the number of genes in common between the three datasets

Three diabetes-related GEO datasets (GSE123658^3^, GSE140627^4^, and GSE143143^5^) are considered for this work (Table 1 and Fig. 4). These datasets comprise patients associated with two distinct groups: patients diagnosed with type 1 diabetes (T1D) and those serving as control subjects (non-T1D). Regarding the demographic data, only the GSE123658 dataset includes information on the gender and age of the patients. This dataset contains 40 female and 42 male patients in total, with a similar gender distribution across the two groups. Specifically, the diabetes group consists of 22 female and 21 male patients, while the control group includes 18 female and 21 male patients. Patient ages range from 19 to 73 years, with an overall median age of 35 years. Among diabetes patients, the median age is 41 years, whereas the control group has a median age of 30 years.

**Table 1:** Number of patients, number of shared genes across different datasets

Dataset	Patients	
	T1D	non-T1D
GSE123658	39	43
GSE140627	5	2
GSE143143	15	15

Regarding the KG embedding implementations, we used an RDF2vec python implementation^6^ and the OpenKE library^7^. The hyperparameters used for each KG embedding model are described in Tables 2 and 3. For RDF2Vec, we used the hyperparemeters defined in [39]. For the remaining KG embedding methods, the default hyperparameters given by OpenKE were used.

**Table 2:** RDF2vec hyperparameters

Hyperparameter	Value
Embedding size	100
Walk depth	4
Maximum number of walks	100

**Table 3:** ComplEx, DistMult, HolE, TransE, TransR hyperparameters

Hyperparameter	ComplEx	DistMult	HolE	TransE	TransR
Embedding size	100	100	100	100	100
Optimization	Adagrad	Adagrad	Adagrad	SGD	SGD
Train times	500	500	500	500	500
Number batches	100	100	100	100	100
Entity neg rate	1	1	1	1	1
Relation neg rate	0	0	0	0	0
Bern	1	1	0	0	0
Alpha	0.5	0.5	0.1	0.001	0.001
Lambda	0.05	0.05	–	–	4
Margin	–	–	–	1	1

The experiments were conducted on a machine equipped with an Intel(R) Xeon(R) processor and 768GB of RAM. The machine was configured to run on AlmaLinux 9.4.

### Evaluation metrics

The evaluation of patient representations in the study is conducted on two dimensions: the diabetes prediction performance and the distribution and clustering of patient representations.

To quantitatively assess the utility of the patient representations, diabetes prediction is formulated as a binary classification task, where the goal is to categorize a set of patients based on whether they have diabetes or not. Therefore, the patient representations are fed into a multi-layer perception (MLP) algorithm for training. To assess the efficacy of the proposed methodology, we analyse the classification performance using four metrics: precision, recall, F1-score, and weighted average F1-score. These metrics enable a comprehensive assessment of the model’s performance, providing a clearer picture of how well the learned representations support diabetes prediction.

To gain deeper insights into the distribution and clustering of different patient representations, we employ t-SNE [40], a statistical method for visualizing high-dimensional data. By projecting the patient embeddings into a two-dimensional space, we can visually observe how well the learned representations capture two clusters, one with diabetic and one with non-diabetic patients.^8^ To quantitatively assess the quality of these clusters, we further compute over the original embeddings a set of clustering evaluation metrics: Calinski-Harabasz score [41], Davies-Bouldin score [42], and silhouette score [43]. Calinski-Harabasz score evaluates the ratio of the sum of between-cluster dispersion and of within-cluster dispersion, providing a measure of cluster separation. A higher score indicates that the clusters are well-separated. Davies-Bouldin score captures the average similarity measure of each cluster with its most similar cluster. A lower Davies-Bouldin score suggests a better clustering. Silhouette score measures how similar each patient is to its own cluster compared to other clusters. The score ranges from −1 to 1, where a higher value indicates better-defined clusters, with points well-matched to their own cluster and poorly matched to neighboring clusters.

### Diabetes prediction

To assess the efficacy of the proposed methodology, we analysed the diabetes performance on the GSE123658 dataset by enriching the training data with information from the GSE140627 and GSE143143 datasets. The GSE123658 dataset was selected due to its suitability for creating a test set of adequate size.

Since our approach involves integrating data from multiple expression datasets into a KG, we compare it against two baselines that employ the expression values of the patient directly as input for the classifier. The first baseline exclusively employs data from GSE140627 for training the classifier. The second baseline represents a more simplistic approach to adding information from other datasets. It involves merging all measured genes across datasets and setting the value to 0 when the patient does not have an expression value.

Furthermore, we compare the proposed methodology with two established frameworks for omics data integration: SIMBA^9^ and MultiMAP^10^. These frameworks were selected for their ability to integrate multiple gene expression datasets and the availability of Python implementations. For a fair comparison, the embedding dimensions for both frameworks were set to 100, consistent with the dimensions used in the KG embedding methods. The embeddings generated by these frameworks were subsequently used to train a classifier. Additionally, we also compare our methodology to a variant that employs a GNN with random initialization, instead of relying on KG embedding methods, followed by training a classifier. For the GNN input, we adopted the patient-gene links strategy to build the gene expression KG, where a link between a patient and a gene is created when the patient’s expression value for that gene is higher than the average expression value calculated either per patient or per gene.

**Fig. 5:**
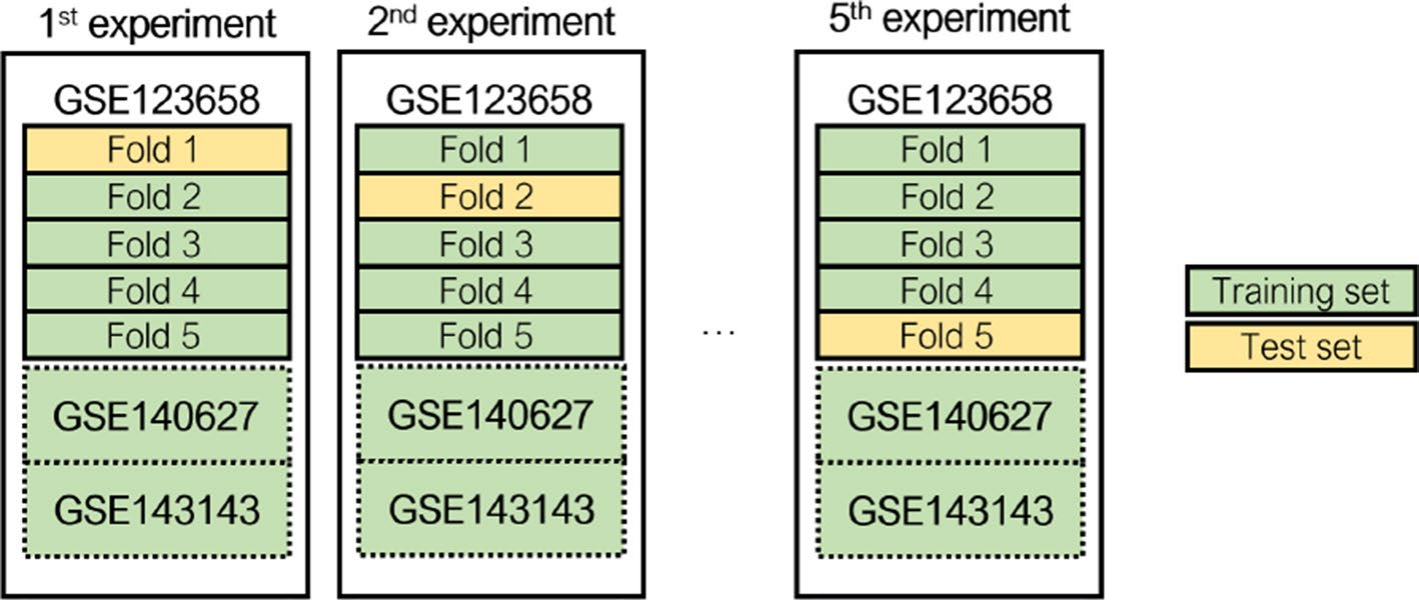
Experimental strategy to split the GSE123658 dataset and enrich with data from the GSE140627 and GSE143143 datasets

We employed a stratified cross-validation strategy to ensure robust evaluation, dividing the GSE123658 dataset into five folds. The same five folds were used throughout all experiments. The reported results represent the average performance over these five folds. Figure 5 illustrates the employed cross-validation strategy.

Table 4 shows the accuracy, precision, recall, f-measure, and weighted average f-measure for the baselines and the proposed methodology. The two baseline results using gene expression values directly indicate that adding information in a simple way from other datasets already slightly enhances performance. This outcome is unexpected since the integration of information from diverse datasets is lacking. However, the datasets have some genes in common (Fig. 4), which can explain that it is still possible to have some advantage in adding more examples in the training set. However, by integrating the information from other datasets in a KG, it becomes evident that training a model with diverse datasets can improve significantly the performance of the machine learning models in all metrics. For example, the best WAF in the gene expression baselines is 0.771, while our approach, yields up to 0.870. Therefore, it confirms our hypothesis that injecting other expression datasets can improve the performance of machine learning models. However, there are performance variations depending on the embedding method and representation strategy used in our approach. With respect to the remaining baselines, the omics integration frameworks that do not incorporate domain-specific knowledge underperform compared to directly using gene expression values. This suggests that dimensionality reduction has a significant performance impact. The lower performance of GNN can be attributed to their challenges in handling the heterogeneous structure of KGs and the lack of node features in our datasets.

Comparing the embedding methods, RDF2vec consistently achieves superior results across a wide range of representation strategies, particularly successful when paired with patient-gene links approaches. This success can be attributed to key aspects of RDF2vec, such as its use of random walks, which allow it to capture long-distance relationships within the KG. In the context of gene expression KGs, this is particularly important, as much of the relevant information resides within the ontology and is not directly attributed to the gene expression at hand, but indirectly connected via multiple hops. The ability of RDF2vec to learn such indirect connections helps to capture the relationships between different genes. These results also align with previous studies, which have demonstrated that RDF2vec is well-suited for several biomedical applications, given its capability to handle complex biomedical data [44]. In contrast, translational methods, such as TransE and TransR, generally perform less effectively, although TransR shows an improvement when combined with a patient-gene links approach. Semantic matching methods-specifically ComplEx, distMult, and HoLE-demonstrate comparable performance values, with HoLE showing a slight advantage when used with the patient-gene links approach.

Regarding the representation strategies, the performance results in Table 4 indicate that using patient-gene links for patient representation is particularly effective, consistently outperforming other approaches. The second-best method, employing the weighted average of gene embeddings for patient representation, also improves performance over some metrics compared to the baselines. Conversely, the binning approach performs the worst, with results often falling below baseline metrics. The binning approach represents gene expression values for individual patients through the creation of blank nodes in the KG. Each blank node corresponds to the expression value of a specific gene for a particular patient. In the KG, this is translated into connecting a patient to the blank node, which is then linked to a gene and the bin that reflects the expression value. Consequently, genes and their expression values are represented as separate triples, which can limit the effectiveness of embedding methods. For instance, walk-based embedding techniques may struggle because the absence of paths connecting genes with their expression values, potentially leading to suboptimal performance in downstream tasks. Interestingly, prior studies suggested that the weighted average of gene embeddings was more successful for diabetes prediction [39], potentially due to the use of datasets with fewer genes. In the current experiments, however, this approach underperformed, likely because the larger gene count reduced its effectiveness. Thus, the gene quantity appears to impact the performance of this representation strategy significantly.

**Table 4:** Mean and standard deviation for accuracy, precision, recall, f1-score, and weighted average f1-score (Acc, Pr, Re, F1, WAF), comparing several approaches. These include our methodology coupled with different embedding methods and different representation strategies, a variant employing a GNN, the gene expression baselines (GE when only the GSE123658 dataset is used and GE+ if the three datasets are used), and the omics integration baselines (SIMBA and MultiMAP). The bold value indicates the highest performance within each embedding method, while the italic value highlights the highest performance for each metric

Metric	Embedding method		Composite gene embeddings			Direct patient embeddings				
	Type	Model	not norm	pat-norm	gene-norm	Binning approach			Patient-gene links approach	
						not norm	pat-norm	gene-norm	pat avg	gene avg
Acc	Walk-based	RDF2vec	0.695 ± 0.041	0.695 ± 0.041	0.707 ± 0.050	0.487 ± 0.238	0.564 ± 0.152	0.561 ± 0.119	**0.879** ± 0.118	0.854 ± 0.060
	Semantic-based	ComplEx	0.671 ± 0.090	0.671 ± 0.090	**0.755** ± 0.090	0.510 ± 0.098	0.524 ± 0.046	0.510 ± 0.125	0.633 ± 0.102	0.512 ± 0.115
		distMult	0.696 ± 0.065	0.696 ± 0.065	**0.780** ± 0.085	0.512 ± 0.100	0.574 ± 0.032	0.488 ± 0.119	0.454 ± 0.101	0.512 ± 0.161
		HolE	0.647 ± 0.068	0.647 ± 0.068	0.721 ± 0.080	0.499 ± 0.052	0.598 ± 0.136	0.403 ± 0.065	0.621 ± 0.078	**0.830** ± 0.042
	Translational	TransE	0.524 ± 0.023	0.499 ± 0.034	0.524 ± 0.023	0.512 ± 0.031	0.487 ± 0.031	0.524 ± 0.023	0.512 ± 0.129	**0.649** ± 0.110
		TransR	0.524 ± 0.023	0.499 ± 0.034	0.499 ± 0.034	0.426 ± 0.083	0.440 ± 0.032	0.414 ± 0.075	0.706 ± 0.065	**0.743** ± 0.047
	GNN								0.414 ± 0.096	**0.478** ± 0.106
	GE		0.768 ± 0.120							
	GE+		0.780 ± 0.083							
	SIMBA		0.559 ± 0.090							
	MultiMAP		0.535 ± 0.091							
Pr	Walk-based	RDF2vec	0.772 ± 0.137	0.772 ± 0.137	0.718 ± 0.048	0.424 ± 0.318	0.558 ± 0.172	0.517 ± 0.170	**0.923** ± 0.154	0.844 ± 0.051
	Semantic-based	ComplEx	0.743 ± 0.168	0.743 ± 0.168	**0.756** ± 0.080	0.483 ± 0.094	0.519 ± 0.094	0.480 ± 0.112	0.654 ± 0.177	0.488 ± 0.174
		distMult	0.762 ± 0.148	0.762 ± 0.148	**0.789** ± 0.088	0.482 ± 0.111	0.629 ± 0.189	0.448 ± 0.124	0.412 ± 0.138	0.519 ± 0.184
		HolE	0.717 ± 0.167	0.717 ± 0.167	0.710 ± 0.088	0.487 ± 0.053	0.566 ± 0.128	0.353 ± 0.086	0.606 ± 0.057	**0.839** ± 0.036
	Translational	TransE	0.000 ± 0.000	0.087 ± 0.175	0.000 ± 0.000	0.094 ± 0.188	0.182 ± 0.223	0.000 ± 0.000	0.477 ± 0.110	**0.797** ± 0.221
		TransR	0.000 ± 0.000	0.087 ± 0.175	0.087 ± 0.175	0.412 ± 0.083	0.372 ± 0.082	0.367 ± 0.085	**0.746** ± 0.136	0.732 ± 0.050
	GNN								0.381 ± 0.109	**0.439** ± 0.162
	GE		0.821 ± 0.141							
	GE+		0.856 ± 0.084							
	SIMBA		0.538 ± 0.124							
	MultiMAP		0.447 ± 0.257							
Re	Walk-based	RDF2vec	0.586 ± 0.158	0.586 ± 0.158	0.636 ± 0.118	0.371 ± 0.300	0.486 ± 0.140	0.432 ± 0.213	**0.875** ± 0.158	0.843 ± 0.102
	Semantic-based	ComplEx	0.564 ± 0.097	0.564 ± 0.097	0.711 ± 0.169	0.536 ± 0.136	0.461 ± 0.123	0.536 ± 0.193	**0.539** ± 0.146	0.404 ± 0.160
		distMult	0.614 ± 0.139	0.614 ± 0.139	**0.743** ± 0.113	0.464 ± 0.176	0.461 ± 0.166	0.464 ± 0.193	0.386 ± 0.139	0.539 ± 0.094
		HolE	0.536 ± 0.111	0.536 ± 0.111	0.718 ± 0.093	0.518 ± 0.109	0.614 ± 0.210	0.357 ± 0.162	0.618 ± 0.172	**0.793** ± 0.104
	Translational	TransE	0.000 ± 0.000	0.200 ± 0.400	0.000 ± 0.000	0.200 ± 0.400	0.400 ± 0.490	0.000 ± 0.000	**0.521** ± 0.209	0.393 ± 0.170
		TransR	0.000 ± 0.000	0.200 ± 0.400	0.200 ± 0.400	0.411 ± 0.049	0.307 ± 0.125	0.382 ± 0.190	0.636 ± 0.118	**0.739** ± 0.125
	GNN								0.382 ± 0.172	**0.407** ± 0.211
	GE		0.671 ± 0.165							
	GE+		0.671 ± 0.200							
	SIMBA		0.432 ± 0.142							
	MultiMAP		0.625 ± 0.371							
F1	Walk-based	RDF2vec	0.639 ± 0.050	0.639 ± 0.050	0.667 ± 0.078	0.390 ± 0.296	0.516 ± 0.152	0.462 ± 0.199	**0.878** ± 0.105	0.842 ± 0.072
	Semantic-based	ComplEx	0.625 ± 0.062	0.625 ± 0.062	**0.724** ± 0.123	0.506 ± 0.110	0.470 ± 0.080	0.501 ± 0.142	0.579 ± 0.127	0.431 ± 0.154
		distMult	0.657 ± 0.047	0.657 ± 0.047	**0.761** ± 0.087	0.464 ± 0.131	0.491 ± 0.077	0.450 ± 0.156	0.397 ± 0.137	0.521 ± 0.128
		HolE	0.590 ± 0.035	0.590 ± 0.035	0.710 ± 0.074	0.492 ± 0.039	0.582 ± 0.153	0.348 ± 0.119	0.598 ± 0.095	**0.811** ± 0.062
	Translational	TransE	0.000 ± 0.000	0.122 ± 0.243	0.000 ± 0.000	0.128 ± 0.256	0.250 ± 0.306	0.000 ± 0.000	0.492 ± 0.154	**0.504** ± 0.156
		TransR	0.000 ± 0.000	0.122 ± 0.243	0.122 ± 0.243	0.407 ± 0.055	0.331 ± 0.108	0.368 ± 0.126	0.671 ± 0.073	**0.728** ± 0.063
	GNN								0.367 ± 0.134	**0.410** ± 0.161
	GE		0.730 ± 0.135							
	GE+		0.729 ± 0.130							
	SIMBA		0.475 ± 0.135							
	MultiMAP		0.489 ± 0.247							
WAF	Walk-based	RDF2vec	0.683 ± 0.033	0.683 ± 0.033	0.702 ± 0.052	0.473 ± 0.246	0.559 ± 0.151	0.546 ± 0.130	**0.870** ± 0.129	0.854 ± 0.060
	Semantic-based	ComplEx	0.662 ± 0.086	0.662 ± 0.086	**0.751** ± 0.094	0.509 ± 0.098	0.515 ± 0.040	0.506 ± 0.122	0.625 ± 0.104	0.499 ± 0.114
		distMult	0.685 ± 0.063	0.685 ± 0.063	**0.779** ± 0.085	0.506 ± 0.099	0.553 ± 0.014	0.483 ± 0.121	0.449 ± 0.105	0.505 ± 0.167
		HolE	0.636 ± 0.060	0.636 ± 0.060	0.719 ± 0.079	0.490 ± 0.060	0.594 ± 0.135	0.389 ± 0.064	0.613 ± 0.081	**0.829** ± 0.043
	Translational	TransE	0.361 ± 0.027	0.333 ± 0.037	0.361 ± 0.027	0.348 ± 0.035	0.320 ± 0.034	0.361 ± 0.027	0.508 ± 0.132	**0.619** ± 0.119
		TransR	0.361 ± 0.027	0.333 ± 0.037	0.333 ± 0.037	0.422 ± 0.083	0.425 ± 0.043	0.407 ± 0.070	0.698 ± 0.065	**0.740** ± 0.047
	GNN								0.396 ± 0.091	**0.461** ± 0.110
	GE		0.764 ± 0.121							
	GE+		0.771 ± 0.093							
	SIMBA		0.549 ± 0.096							
	MultiMAP		0.430 ± 0.150							

### Distribution and clustering of patient representations

**Fig. 6:**
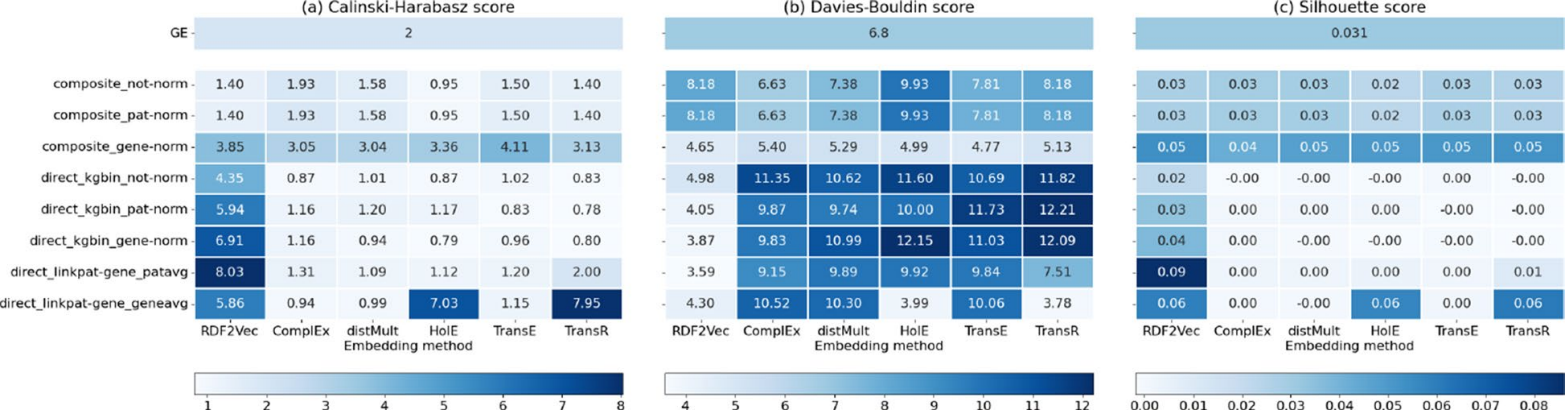
Heat maps depicting the values for three clustering metrics - **a** Calinski-Harabasz score (higher is better), **b** Davies-Bouldin score (lower is better), **c** Silhouette score (higher is better). Each heat map shows the clustering metric values with the x-axis representing different embedding methods (RDF2Vec, ComplEx, distMult, HolE, TransE, TransR) and the y-axis representing different strategies for generating patient representation using the embeddings (composite_not-norm, composite_pat-norm, composite_gene-norm, direct_kgbin_not-norm, direct_kgbin_pat-norm, direct_kgbin_gene-norm, direct_linkpat-gene_patavg, direct_linkpat-gene_geneavg). At the top of each heat map, the clustering metric value is provided for patient representations derived directly from gene expression data (GE) as a baseline point

Figure 6 presents heatmaps displaying the values of three clustering metrics (Calinski-Harabasz score, Davies-Bouldin score, and silhouette score) applied to two patient labels: control and disease. The values are computed for patients across the three datasets (GSE123658, GSE140627, GSE143143). Each metric evaluates clustering quality differently: higher Calinski-Harabasz and silhouette scores indicate better clustering, whereas, for the Davies-Bouldin score, lower values suggest stronger clustering performance. Despite these differences, the observed patterns across the metrics are largely consistent. Analyzing the x-axis, which represents various KG embedding methods, three methods - RD2Vec, HolE, and TransR - consistently show superior clustering performance. In contrast, Complex, DistMult, and TransE yield similar but somewhat worse results. The y-axis shows patient representation strategies. Here, a distinction is generally seen between patient representations created by a weighted average of gene embeddings (composite_not-norm, composite_pat-norm, composite_gene-norm) and those generated directly through embeddings in the KG. Notably, RDF2vec deviates from this pattern, achieving better clustering performance across a broader range of representation strategies. Specifically, RDF2vec achieves the best metric values when the KG includes patient-gene links when gene expression exceeds the average expression level for each patient (direct_linkpat-gene_patavg). The clustering performance of RDF2vec may be attributed to its path-based approach, which effectively captures entity relationships and avoids the challenges that translational and semantic matching methods encounter with learning entity representations, rather than ontology class representations.

Another interesting aspect involves comparing clustering metrics between patient representations generated using the gene expression KG and those based directly on gene expression values. Most of the time, composite representations that employ gene embeddings or representations obtained with RDF2vec outperform the baseline approaches in clustering performance.

**Fig. 7:**
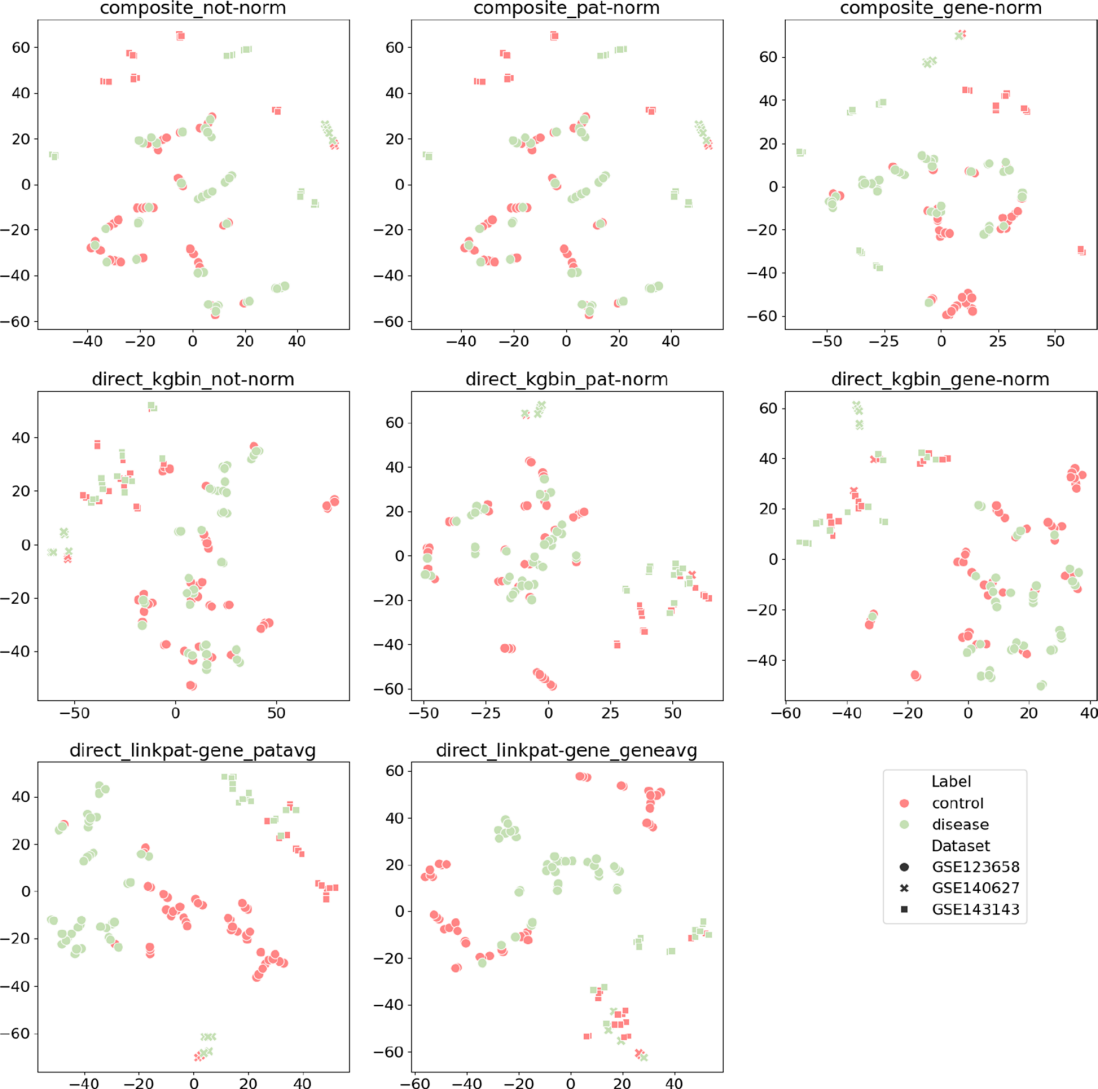
Plots illustrating patient representations derived from different strategies using embeddings (composite_not-norm, composite_pat-norm, composite_gene-norm, direct_kgbin_not-norm, direct_kgbin_pat-norm, direct_kgbin_gene-norm, direct_linkpat-gene_patavg, direct_linkpat-gene_geneavg) are presented. For each strategy, patient representations were reduced to two dimensions using the t-SNE technique. Each point corresponds to a patient, with the color representing their label (e.g., control or disease) and the shape denoting the dataset of origin. Effective separation of colors in the plots indicates successful differentiation between the two labels, while clustering of points by shape suggests a bias in the patient representations based on their dataset of origin

In addition to evaluating the clustering patterns of patients labeled as disease and control, it is also insightful to examine how patient embeddings from various datasets are being represented within the semantic space depending on the strategies of representation. Figure 7 visualizes the embeddings obtained with RDF2vec using t-SNE across different representation strategies. Comparing these plots reveals distinct representation behaviors. For example, strategies like composite_gene-norm and direct_kgbin_pat-norm seem to effectively align patients from different datasets within the same semantic space. In contrast, strategies such as direct_kgbin_gene-norm clearly delineate three groups, each corresponding to a distinct dataset. Notably, the plot using direct_linkpat-gene_patavg strategy aligns with findings in Fig. 6, showing increased separation between disease and control, while patients remain generally clustered by dataset, albeit with some overlap.

Finally, it is essential to compare the evaluation through embedding visualizations and clustering metrics (Figs. 6 and 7) with the diabetes performance evaluation (Table 4). While the patient-gene links strategy yielded label separation primarily when combined with RDF2vec embeddings, incorporating machine learning extended this success across other embedding methods as well.

## Conclusion

Several approaches for diabetes prediction rely on the analysis of expression data, which provide a detailed molecular profile reflecting gene activity and regulation and therefore can uncover relationships between specific genes and the development of diabetes. However, exploring expression data in machine learning presents its own set of challenges. Existing expression datasets related to diabetes have a very low number of patients, which can be a limitation for data-driven methods such as machine learning algorithms. Therefore, the integration of multiple expression datasets can address the issue of limited patients and, at the same time, offer a comprehensive perspective on the complex factors influencing diabetes. However, a significant hurdle arises since different datasets measure the expression of different genes. Not only do they often capture expression for distinct sets of genes, but even when they overlap, the experimental conditions under which these genes were measured might differ substantially. These differences render the features extracted from different datasets incompatible, making the integration process harder.

We have developed an approach that enables a comprehensive representation of gene expression data from different datasets within a KG. Through semantic links and domain-specific knowledge, KGs can create a unified knowledge space to connect datasets from distinct studies. In this work, we have explored different strategies to include the expression data in the KG and different strategies to represent the patients within the KG using KG embedding methods. The results of our experiments showed that integrating gene expression data in a KG is able to improve the performance of diabetes prediction.

The proposed approach is versatile and can be extended to the prediction of other diseases. The core steps - such as data preprocessing, patient representation, and predictive modeling - are not disease-specific and can be inherently applicable, as long as a gene expression dataset is available and the objective involves predicting disease presence or absence for a patient. In future research, it will be crucial not only to incorporate diverse datasets with richer demographic details to further validate our findings but also to apply and assess the methodology in the context of other diseases beyond diabetes.

In addition, there are also some limitations of the proposed approach that can be addressed in future work. One limitation is the potential integration of multimodal data. Currently, the KG only incorporates gene expression data, but incorporating other types of omics data (e.g., proteomics, metabolomics) or even clinical data could offer a more holistic view of diseases. Another limitation is the formulation of the disease prediction task, which is cast as a binary classification problem. This approach might oversimplify the complexity of prediction for some diseases. Therefore, expanding this methodology to support multi-class or multi-label classification would allow the model to better capture the complexities of disease, such as distinguishing between different stages of a disease (e.g., cancer stages) or identifying various subtypes of a disease (e.g., different cancer types).

## Data Availability

The datasets used to support the results of this manuscript are available on Gene Expression Omnibus (GEO) database.

## Footnotes

1 https://www.uniprot.org/id-mapping.

2 https://www.w3.org/TR/owl2-mapping-to-rdf/.

3 https://www.ncbi.nlm.nih.gov/geo/query/acc.cgi?acc=GSE123658.

4 https://www.ncbi.nlm.nih.gov/geo/query/acc.cgi?acc=GSE140627.

5 https://www.ncbi.nlm.nih.gov/geo/query/acc.cgi?acc=GSE143143.

6 https://github.com/IBCNServices/pyRDF2vec.

7 https://github.com/thunlp/OpenKE/tree/OpenKE-Tensorflow1.0.

8 It is important to note that we do not perform clustering on the data, but exploit *metrics* that are used for measuring the separation of clusters in clustering to understand which representation and embedding methods provide the best class separation of diabetic and non-diabetic patients.

9 https://simba-bio.readthedocs.io.

10 github.com/Teichlab/MultiMAP.

## Code availability

The source code for the proposed methodology is available on GitHub: https://github.com/ritatsousa/expressionKG.
